# Assessment Tools and Psychosocial Consequences of Smartphone Addiction in Nursing Students: A Systematic Review and Meta-Analysis

**DOI:** 10.3390/healthcare13202639

**Published:** 2025-10-20

**Authors:** María Dolores Lazo-Caparrós, José Luis Gómez-Urquiza, Ana González-Díaz, Inmaculada Pérez-Conde, Piedad Gómez-Torres, María José Membrive-Jiménez

**Affiliations:** 1 Instituto Nacional de Gestión Sanitaria, Hospital Universitario de Ceuta, 51003 Ceuta, Spain; mdlazo@correo.ugr.es; 2 Faculty of Health Sciences of Ceuta, University of Granada, 51005 Ceuta, Spain; agonzalezd@ugr.es (A.G.-D.); inmaconde@ugr.es (I.P.-C.); piedadgomez@ugr.es (P.G.-T.); mjmembrive@ugr.es (M.J.M.-J.)

**Keywords:** smartphone addiction, nursing students, systematic review, meta-analysis, clinical decision-making, nomophobia

## Abstract

**Background/Objectives**: Problematic smartphone use is common among nursing students and has been linked to academic and psychosocial difficulties. This PROSPERO-registered systematic review (CRD42024559668) identified the instruments used to assess smartphone addiction in nursing students and, secondarily, pooled typical addiction levels using the Smartphone Addiction Scale–Short Version (SAS-SV; 10–60) and examined psychosocial correlates. **Methods**: Following PRISMA 2020, we searched PubMed, Scopus, Web of Science, CINAHL and ScienceDirect from 1 January 2014 to 9 May 2024. Eligible studies assessed problematic smartphone use in undergraduate nursing students with validated instruments, while development-only studies and pandemic-specific contexts were excluded. Methodological quality was appraised using the JBI checklist, and a random-effects meta-analysis was performed to estimate pooled scores and explore cross-study variability. **Results**: Fifty-three studies met inclusion; eleven contributed to the SAS-SV meta-analysis (N = 5586). The pooled mean score was 29.5 (95% CI 27.7–31.3), with very high heterogeneity (I^2^ = 98%). Sensitivity analyses yielded similar results, and no publication bias was detected. Across studies, higher smartphone addiction was correlated with elevated stress and anxiety, sleep disturbance, and poorer academic and clinical performance. **Conclusions**: Nursing students’ SAS-SV scores cluster around ~29/60, with substantial between-study variability. Higher addiction scores were consistently associated with stress, anxiety, poor sleep, and reduced academic and clinical performance. However, interpretation is limited by the cross-sectional nature of the included studies and the very high heterogeneity observed. Standardising measurement is essential, but equally important is developing targeted educational interventions to foster healthier smartphone habits in nursing education. These results may guide nursing educators and institutions to design programs that foster healthier digital habits and support students’ academic and clinical performance.

## 1. Introduction

Over the past decade, smartphone use has grown exponentially, becoming an indispensable tool in the daily lives of millions of people worldwide, including university students in the health sciences [1]. Its use in educational contexts has shown potential benefits, such as rapid access to clinical information, enhanced communication with healthcare teams, and support for assisted decision-making [2,3]. Nevertheless, these advantages coexist with growing evidence that problematic or uncontrolled smartphone use may lead to cognitive, emotional, and behavioural difficulties that directly affect learning and clinical training [4].

One of the most studied emerging phenomena is nomophobia, defined as the irrational fear of being without a mobile phone [5,6]. This condition, along with smartphone addiction, has been consistently associated with elevated anxiety, academic stress, and dysfunctional decision-making styles, such as avoidance and procrastination, particularly in healthcare students [7,8]. In addition, research has reported associations with emotional dysregulation, reduced sleep quality, poorer academic performance, impaired empathy, and diminished interpersonal communication during clinical training, as well as behaviours such as cyberloafing and phubbing, which can interfere with learning efficacy and the quality of professional relationships [1,9,10,11,12,13].

Several narrative reviews and broader systematic reviews have previously explored problematic smartphone use in university students, highlighting its prevalence and psychosocial impact [4,14]. For instance, Busch & McCarthy [4] provided a general overview of antecedents and consequences, while Candussi et al. [14] synthesised prevalence and patterns among undergraduates. However, specific evidence in nursing education remains fragmented, with no prior meta-analytic synthesis of addiction levels and related outcomes.

This study therefore addresses a critical gap by providing a systematic review and meta-analysis exclusively focused on nursing students. By unifying data on assessment tools, pooled addiction scores, and psychosocial correlates, it contributes an original and rigorous synthesis that has not previously been available to guide nursing education and clinical training.

Despite these concerns, there is considerable resistance among students to accepting restrictive policies on smartphone use during clinical practice. Cho and Lee [2] found that while over 46% of respondents considered smartphone use inappropriate in clinical settings, only 29% supported formal restrictive regulations. Beyond the academic context, problematic smartphone use is particularly relevant in clinical environments, where it has been linked to reduced quality of learning, lower perceived social support, and even potential risks for patient care [14,15,16].

Despite the growing body of literature, a major methodological challenge is the lack of standardisation across the instruments used to assess smartphone addiction. Multiple scales with different structures and cut-off values coexist, limiting comparability across studies and hindering the development of unified educational and clinical responses [17,18].

Therefore, the primary objective of this systematic review is to describe and synthesise the validated instruments used to assess problematic smartphone use among nursing students. Secondary objectives are to (1) identify the most frequently used tool, (2) conduct a meta-analysis of studies using the most common instrument (SAS-SV) to estimate average addiction levels, and (3) summarise the main psychological, academic, and clinical correlates reported across studies.

Unlike previous narrative reviews, this study provides an updated and quantitative synthesis of smartphone addiction levels and their academic, clinical, and psychological consequences among nursing students. In addition, it seeks to promote greater consistency in the measurement of this construct by unifying the use of assessment tools in the field, thereby reducing current methodological fragmentation and contributing to a more robust framework for evaluating this addictive behaviour in nursing education.

## 2. Materials and Methods

This review followed PRISMA 2020 [19] and was registered in PROSPERO (CRD42024559668).

### 2.1. Study Design

We conducted a systematic review with an embedded meta-analysis. The primary aim was to identify and describe validated instruments used to assess smartphone addiction or problematic smartphone use among undergraduate nursing students, focusing on instrument structure, scoring, and psychometric validation. The secondary aim was to synthesise mean scores using the most frequently employed and psychometrically homogeneous tool—the Smartphone Addiction Scale–Short Version (SAS-SV; score range 10–60).

### 2.2. Information Sources and Search Strategy

A systematic search was performed across five databases: PubMed, Scopus, Web of Science, CINAHL, and ScienceDirect. The search covered literature published from 1 January 2014 to 9 May 2024, encompassing the most recent decade of research on smartphone addiction in nursing education.

Search terms combined free-text keywords and Boolean operators tailored to each database’s syntax. The strategy aimed to retrieve studies involving nursing students and focused on mobile or smartphone addiction and associated assessment tools. Reference lists of included studies were also screened manually. No grey literature was included.

The search strings were as follows:

PubMed: (“smartphone addiction” OR “mobile phone addiction” OR “cell phone addiction”) AND (“nursing students” OR “student nurses”) AND (“measurement instrument” OR “assessment tool” OR “scale” OR “questionnaire”)

Scopus: TITLE-ABS-KEY((“nursing students” OR “student nurses”) AND (“smartphone addiction” OR “mobile phone addiction” OR “cell phone addiction”) AND (“assessment” OR “evaluation” OR “instrument” OR “questionnaire”))

Web of Science: TOPIC: (“smartphone addiction” OR “mobile phone addiction” OR “cell phone addiction”) AND (“nursing students” OR “student nurses”) AND (“measurement instrument” OR “assessment tool” OR “scale” OR “questionnaire”)

CINAHL: (“smartphone addiction” OR “mobile phone addiction” OR “cell phone addiction”) AND (“nursing students” OR “student nurses”) AND (“measurement instrument” OR “assessment tool” OR “scale” OR “questionnaire”)

ScienceDirect: (“smartphone addiction” OR “mobile phone addiction” OR “cell phone addiction”) AND (“nursing students” OR “student nurses”) AND (“measurement instrument” OR “assessment tool” OR “scale” OR “questionnaire”)

Reference lists of included articles were also screened manually to identify additional relevant studies. The search returned a total of 229 records before de-duplication, distributed as follows: CINAHL (*n* = 84), ScienceDirect (*n* = 46), Web of Science (*n* = 43), Scopus (*n* = 29), and PubMed (*n* = 27). The last search was conducted on 9 May 2024.

### 2.3. Eligibility Criteria

We included quantitative, analytical cross-sectional studies involving pre-licensure nursing students. Studies with mixed-discipline samples were eligible only if data specific to nursing students could be extracted, and in such cases only the nursing subgroup (*n*, mean, SD) was used for quantitative synthesis. Quasi-experimental studies were eligible provided that baseline (pre-intervention) data were reported separately, which were extracted exclusively for comparability. Eligible studies were required to utilise a validated tool to assess problematic smartphone use and report quantitative scores or prevalence estimates. Only articles published in English or Spanish between January 2014 and May 2024 were included.

We excluded qualitative designs; literature reviews, editorials, or opinion pieces; studies involving patient populations or midwifery-only cohorts and articles using non-validated instruments or lacking quantitative data.

For the meta-analysis, studies that employed the SAS-SV and provided sufficient statistical information (mean, standard deviation, or convertible equivalents) were included. When scores were stratified (e.g., by academic year), group means and SDs were combined into a single estimate. One study that presented an approximated pooled mean without subgroup nursing students was excluded from the main analysis but included in sensitivity analyses [20].

For handling of mixed samples and quasi-experimental designs, when studies included mixed student populations (e.g., nursing and medical) [21], we extracted only the nursing subgroup provided that *n*, mean and standard deviation were reported separately; otherwise, the study was excluded from quantitative synthesis. For quasi-experimental studies, we included baseline (pre-intervention) estimates only, to avoid conflating intervention effects with typical levels, and appraised them using the JBI Checklist for Quasi-Experimental Studies.

We mapped all validated instruments assessing smartphone addiction/problematic use (e.g., SAS/SAS-SV, SPAI, PMPU, SQAPMPU). The quantitative meta-analysis was restricted to SAS-SV (10–60) to ensure metric homogeneity for pooled means; studies using other instruments were synthesised narratively, including their psychosocial correlates (e.g., stress, anxiety, sleep, academic/clinical outcomes).

For sensitivity analyses, we repeated the SAS-SV meta-analysis excluding (i) mixed-sample studies even when subgroup data were available and (ii) the quasi-experimental study and found that the pooled mean and its interpretation remained stable.

A concise summary of the inclusion and exclusion criteria, including the handling of mixed samples and quasi-experimental designs, is provided in Table 1 for clarity and transparency.

### 2.4. Study Selection and Data Extraction

Two reviewers independently screened all titles and abstracts and subsequently evaluated full texts according to the inclusion criteria. Discrepancies were resolved through discussion; a third reviewer was available but not required. Inter-rater reliability was quantified using Cohen’s Kappa coefficient. Agreement between reviewers was 85%, with κ = 0.66, indicating substantial agreement.

Data were extracted using a pre-specified form, including study characteristics (author, year, country, design, sample size, percentage of female participants, age), instruments used (name, scale, scoring, validation), and outcome measures (mean scores and key findings related to smartphone addiction) as shown in Supplementary Table S2.

Of the 15 studies that employed the SAS-SV, 11 provided sufficient information (sample size, mean, and standard deviation) to be included in the quantitative synthesis. Four studies were excluded from the meta-analysis due to incomplete or stratified reporting that precluded reliable pooling, although they were retained in the narrative synthesis.

For studies included in the meta-analysis (SAS-SV only), the following data were extracted: author, year, country, sample size, SAS-SV mean and standard deviation (range 10–60), standard error when available, per-item scores converted to totals (×10), percentage female, academic level, mean age, language of the instrument, response rate, sampling method, and risk-of-bias rating according to the JBI checklist. When needed, SDs were derived from SE or 95% CI using standard formulas. In stratified samples, subgroup means were combined. All decisions followed pre-established rules.

### 2.5. Risk of Bias Assessment

A summary of the risk of bias ratings using the JBI Critical Appraisal Checklist for Analytical Cross-sectional Studies (8 items: Q1–Q8) [22] is in Supplementary Table S3. One quasi-experimental study, Bayir et al. [23], was appraised using the JBI Checklist for Quasi-Experimental Studies, showing low risk of bias despite the lack of blinding and some sample attrition, is flagged as NA in the cross-sectional table.

Two reviewers independently conducted the assessments after piloting the checklist; disagreements were resolved by discussion, and a third reviewer was available but not required. Inter-rater agreement was not quantified, but agreement was high across decisions. Overall risk was judged using a domain-based approach without numerical scoring: studies were rated low risk when most items were met, including identification and management of confounding (Q5–Q6), with no critical flaws in measurement (Q3–Q4) or analysis (Q8); high risk was assigned when several key domains were unmet or unclear, such as invalid exposure or outcome measurement or inappropriate statistical analysis; all other studies were classified as moderate risk.

### 2.6. Data Analysis

The quantitative synthesis was restricted to studies that applied the Smartphone Addiction Scale–Short Version (SAS-SV), as this was the most frequently used and psychometrically homogeneous instrument identified. The primary outcome was the pooled mean SAS-SV score (range 10–60).

Random-effects models with Restricted Maximum Likelihood Estimation (REML) were fitted to account for expected variability between studies. Analyses were conducted in Jamovi version 2.6 (MAJOR module; R 4.4, metafor package) [24]. The results were expressed as pooled means with 95% confidence intervals (CIs), accompanied by 95% prediction intervals (PIs) to estimate the range of expected effects in new studies.

Between-study heterogeneity was assessed using Cochran’s Q, Higgins’ I^2^, and τ^2^. Given the very high heterogeneity anticipated, exploratory subgroup analyses were planned by country, year of publication, and validated language version of the SAS-SV. Formal meta-regression was not attempted because the number of included studies (k = 11) was insufficient to support reliable multivariable modelling.

Small-study effects and publication bias were evaluated through visual inspection of funnel plots, Egger’s regression intercept test, and Kendall’s rank correlation (τ). These tests were interpreted cautiously given their low power with fewer than 15 studies.

Standardised data handling rules were applied, specifically: (i) when only per-item means were available, totals were obtained as Mean per item × 10; (ii) when subgroup results were reported, pooled means and SDs were computed using standard formulas; (iii) when only SE or 95% CIs were reported, SDs were derived using established methods (e.g., SD = SE × √*n*; SD = √*n* × (Upper − Lower)/3.92). These procedures were pre-specified and applied consistently across studies. Full details and worked examples of these conversions are provided in Supplementary Table S1.

We did not apply the GRADE framework, as it is primarily designed for intervention studies and is not routinely recommended for continuous outcomes derived from cross-sectional data. Instead, methodological quality was assessed with the Joanna Briggs Institute (JBI) checklist for analytical cross-sectional studies, which is more appropriate for the included designs.

In addition, pre-specified sensitivity analyses were conducted to test the robustness of pooled estimates, including exclusion of studies with approximated pooled means, exclusion of studies requiring item-to-total score conversions, and restriction to studies rated as low risk of bias according to the JBI checklist. All results were consistent in direction and magnitude.

## 3. Results

### 3.1. Search Results and Study Selection Process

**Figure 1:**
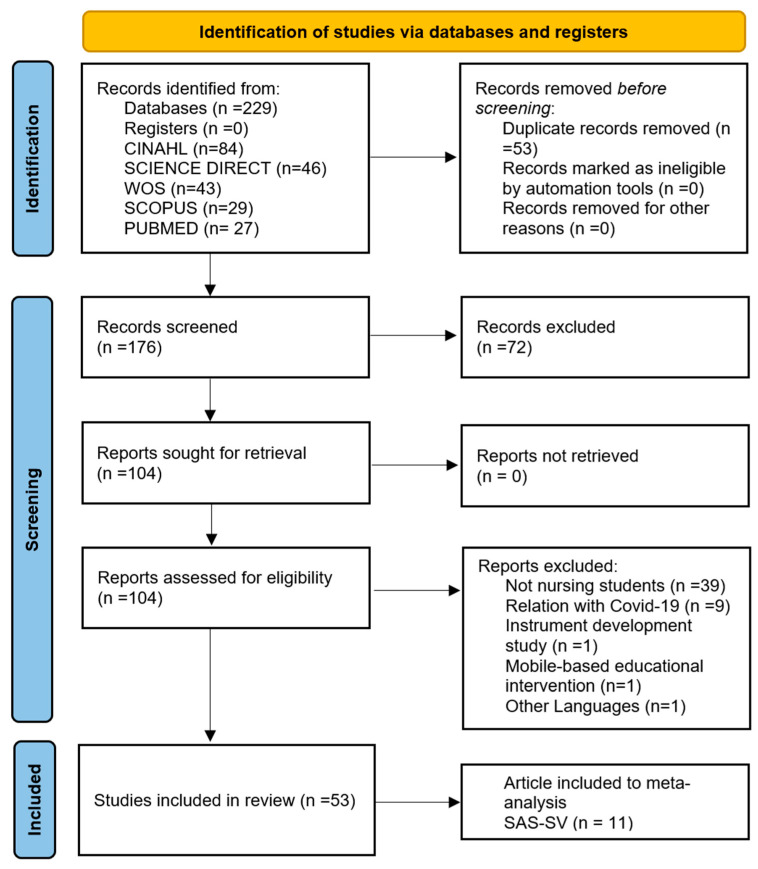
PRISMA flow diagram of the study selection and inclusion process.

A total of 229 records were identified through various databases. Using the Zotero reference manager, 53 duplicates were removed, leaving 176 records for title and abstract screening. Of these, 104 full-text articles were retrieved for detailed evaluation. At this stage, studies were excluded if they did not report nursing students separately from other disciplines (*n* = 39), if they were related to COVID-19 (*n* = 9), if they focused on the development of an instrument (*n* = 1) [25], if they described educational audiovisual content via smartphones (*n* = 1) [26], or if they were published in other languages (*n* = 1). In total, 51 reports were excluded. Finally, 53 studies were included in the systematic review. The selection process is illustrated in Figure 1.

### 3.2. Characteristics of the Included Studies

The 53 studies included in this systematic review represent a broad geographical distribution, with a predominance of research conducted in Turkey, followed by India, South Korea, China, and Spain. This reflects a growing international interest in the study of smartphone addiction among nursing students. Most publications were concentrated between 2018 and 2024, with the peak in 2021, the year with the highest number of studies published.

The largest sample was reported in the study by Zhou [27], which included 1445 nursing students. The most frequently used assessment tool was the Smartphone Addiction Scale–Short Version (SAS-SV), employed in more than one-third of the studies. A concise summary of the included studies is presented in Table 2; a detailed dataset is available in Supplementary Table S2. These characteristics help contextualise the variety of instruments used to assess smartphone addiction and its related constructs.

### 3.3. Assessment Tools Used

In Table S2, the ‘Mean score of instrument(s) used’ column presents the scores of the tools applied to assess problematic smartphone use and the psychosocial variables examined in each study, whereas the ‘Main findings’ column summarises the reported correlations between these variables.

Across the 53 included studies, a wide range of instruments was employed to assess smartphone addiction or problematic use (Table 3). The Smartphone Addiction Scale–Short Version (SAS-SV) was the most frequently applied tool (k = 15), followed by the Nomophobia Questionnaire (NMP-Q; k = 9), the full Smartphone Addiction Scale (SAS; k = 6), the Digital Addiction Scale (DAS; k = 3), and the Smartphone Addiction Inventory (SPAI; k = 2). Other instruments, such as the MPPUS, MPPUS-10, CPAS, MTUAS and PMPUQ, appeared less frequently (k = 1–2).

In summary, the objectives of mapping the instruments used to evaluate problematic smartphone use (Table 3) and documenting their associations with psychosocial variables (Supplementary Table S2) were achieved, providing a comprehensive overview of both the measurement tools and their reported correlations in nursing students.

### 3.4. Psychological Impact

Some studies in this review reported significant associations between smartphone addiction or nomophobia and psychological variables such as stress, anxiety, depression, emotional dysregulation, low self-esteem, impulsivity, and sleep disturbances.

For example, Barzegari et al. [28] found that higher smartphone addiction scores were significantly associated with depressive symptoms, while Mohamed and Mostafa [9] reported a correlation between smartphone addiction and both low self-esteem and depressive states. Similarly, Ghosh et al. [7] and Kalal et al. [1] highlighted that smartphone addiction was related to poor sleep quality, a finding also supported by Çelebi [11] and Uzuncakmak et al. [62].

El-Ashry et al. [35] identified positive correlations between nomophobia and anxiety or impulsivity, whereas Özer et al. [55] reported that emotional dysregulation was a key factor linked to higher smartphone addiction scores. Furthermore, Han et al. [39] discussed the impact of problematic mobile use on stress, emotional exhaustion, and procrastination, with statistically significant associations.

These findings consistently suggest that excessive or problematic smartphone use is negatively associated with key psychological health indicators among nursing students.

### 3.5. Academic Impact

Several studies identified a significant relationship between smartphone addiction or nomophobia and poor academic performance, reduced motivation, and increased procrastination. For instance, Machado et al. [47] and Kalal et al. [1] found that students with high addiction scores reported significantly lower academic achievement. Similarly, Berdida and Grande [29] and Tárrega-Piquer et al. [59] reported that nomophobia was negatively correlated with intrinsic motivation and associated with academic procrastination.

The influence of smartphone use on attention and learning was also addressed. Han et al. [39] and Dayapoğlu et al. [34] observed that problematic use was linked to distraction, reduced concentration, and poorer grades. Savci et al. [56] and Márquez-Hernández et al. [50] further suggested that smartphone overuse was associated with dysfunctional cognitive styles and impaired academic decision-making.

These findings indicate that excessive smartphone use may compromise academic functioning in nursing students by interfering with concentration, academic achievement, and decision-making processes.

### 3.6. Meta-Analysis Results

Of the 53 studies included in the systematic review, 15 studies that employed the SAS-SV, 11 provided complete data (sample size, mean, and standard deviation) and were included in the quantitative synthesis, yielding a pooled sample of 5586 nursing students. The remaining four SAS-SV studies were excluded from the meta-analysis due to incomplete or stratified reporting and are considered in the narrative synthesis. The characteristics of the included studies are detailed in Table 4.

**Figure 2:**
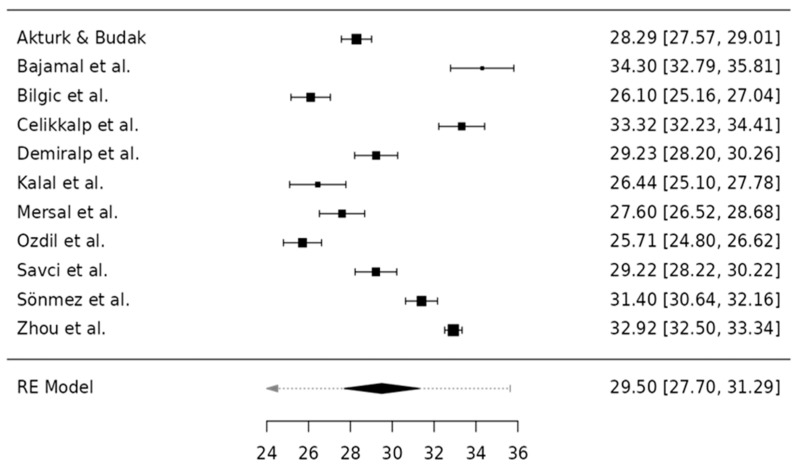
Forest Plot of Mean Scores for the SAS-SV Questionnaire [1,10,12,13,16,21,27,51,54,56,58].

A random-effects meta-analysis was conducted using Jamovi version 2.6 (MAJOR module), applying Restricted Maximum Likelihood Estimation to model between-study variance. The pooled mean SAS-SV score was 29.50 (95% CI 27.70–31.29), indicating a moderate average level of problematic smartphone use among nursing students. This result is illustrated in Figure 2, which presents the forest plot with the mean scores and corresponding confidence intervals for each study.

**Figure 3:**
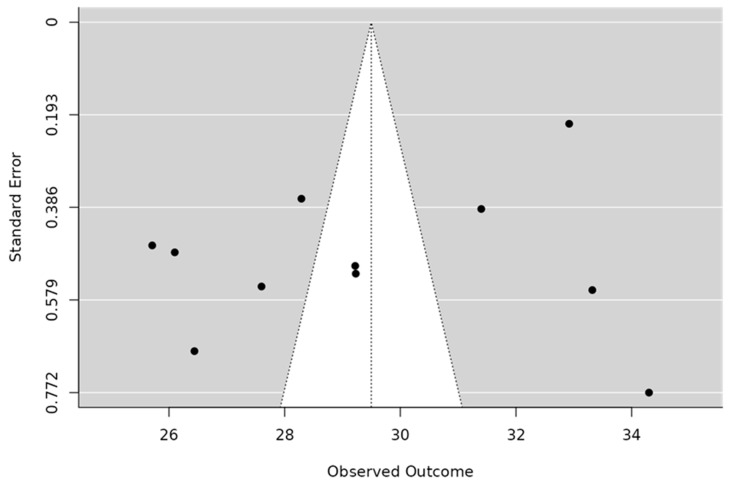
Funnel Plot of Mean Scores for the SAS-SV Questionnaire.

The pooled SAS-SV mean was 29.5 (95% CI 27.7–31.3), with very high heterogeneity (I^2^ = 97.9%, τ^2^ = 8.97; Q(10) = 485.2, *p* < 0.001). The 95% prediction interval (23.4–35.6) indicated that average scores in future comparable studies are expected to fall within this range. Sensitivity analyses excluding studies with approximated means, item-to-total conversions, or higher risk of bias yielded consistent results. Tests for small-study effects showed no evidence of publication bias (Egger’s *p* = 0.982; Kendall’s τ = −0.018, *p* = 0.94) (Table 5). A leave-one-out analysis confirmed the stability of the pooled estimate. The trim-and-fill procedure did not impute missing studies, supporting the robustness of the pooled estimate. The funnel plot is shown in Figure 3.

To assess the stability of the pooled mean, the analysis was cross-validated in SPSS version 29 using a random-effects model with Knapp–Hartung adjustment. This approach yielded a comparable pooled mean of 29.50 (95% CI 27.45–31.55), confirming the consistency of the main estimate. No substantial deviations were found when excluding the study with an approximated pooled mean, removing studies requiring per-item to total score conversion, or restricting to studies rated as low risk of bias. These findings support the robustness and reliability of the meta-analytic conclusions across different analytical approaches and study inclusion scenarios.

Restricting the analysis to studies at low risk of bias (JBI checklist) and excluding those that required conversions yielded pooled estimates consistent in direction and magnitude with the main analysis, supporting the robustness of the findings.

Study means were widely dispersed (≈25.7–34.3 on the SAS-SV; Table 4), consistent with the very high I^2^. The evidence base was geographically skewed (predominantly Turkey), and validated language versions varied across studies, alongside heterogeneous sampling strategies (census, convenience, quota). These features plausibly contributed to between-study variability. Pre-specified subgroup checks by country, year of publication and instrument language did not yield a consistent pattern and did not materially reduce heterogeneity. Given k = 11, we refrained from meta-regression to avoid unstable estimates. Accordingly, the pooled mean should be interpreted with caution, and the prediction interval is emphasised as a more informative summary of expected scores across settings.

**Figure 4:**
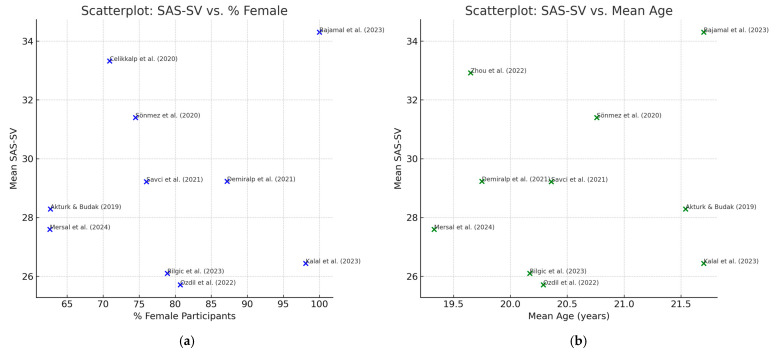
Scatterplots SAS-SV. Note: Exploratory scatterplots of mean SAS-SV scores against (**a**) percentage of female participants and (**b**) mean age of nursing student cohorts [1,10,12,13,16,21,27,51,54,56,58]. Data are derived from the 11 studies included in the meta-analysis (Table 4). Missing values for sex distribution [27] and mean age [21] explain why not all studies are represented in each plot.

To further explore potential sources of heterogeneity, an exploratory scatterplot was generated plotting mean SAS-SV scores against the percentage of female participants and mean age of the cohorts (Figure 4). While no consistent trend was evident, the plots illustrate the wide dispersion of scores across samples with differing demographic profiles.

### 3.7. Risk of Bias Assessment

**Figure 5:**
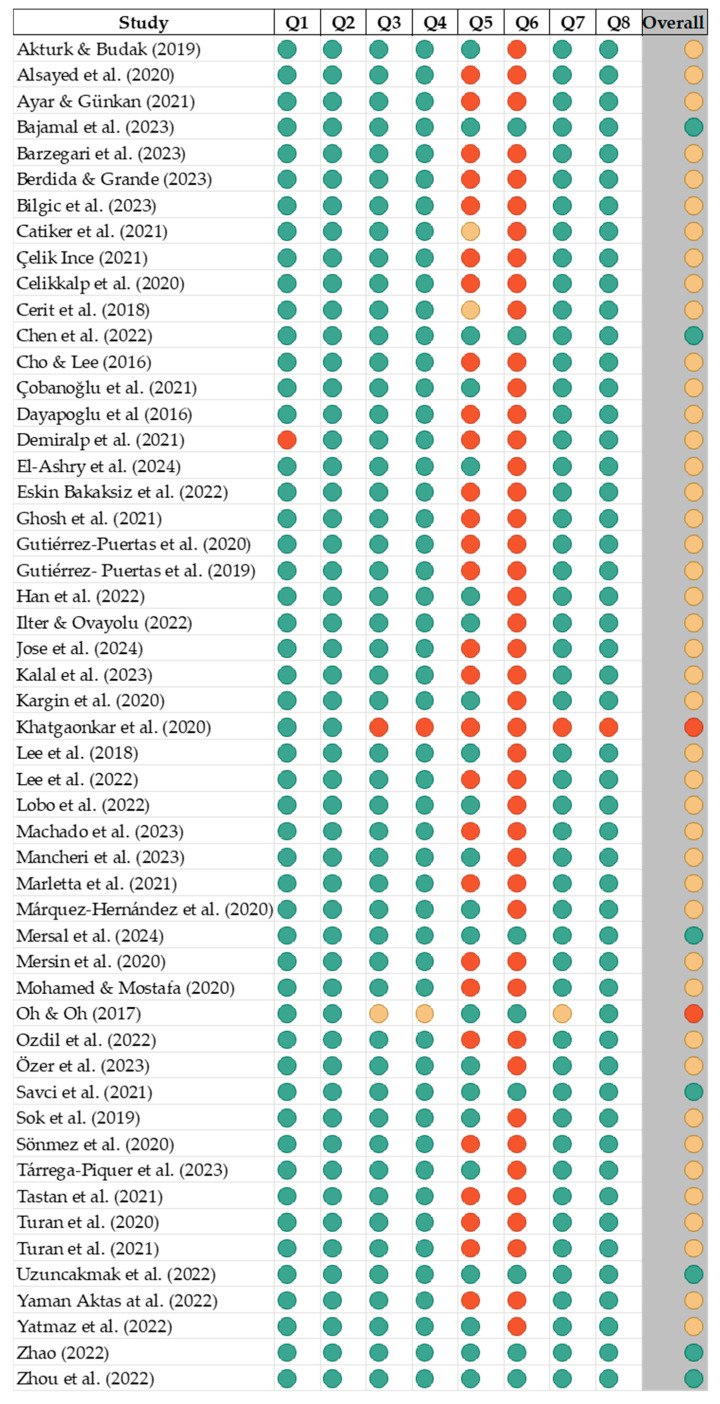
Traffic-light plot JBI. Note: Traffic-light plot generated from JBI checklists. Green = “Yes”, Red = “No”, Yellow = “Unclear” responses for each domain. The “Overall” column represents the global risk-of-bias judgement (Green = Low [13,27,33,51,56,62,64], Yellow = Moderate [1,2,3,7,8,9,10,12,15,16,17,20,21,23,28,29,30,31,32,34,35,36,37,38,39,40,41,42,44,45,46,47,48,49,50,52,54,55,57,58,59,60,61,63], Red = High [43,53]). Figure 4 summarises the risk of bias for the 52 cross-sectional studies included. The quasi-experimental study [23] was appraised separately with the JBI Checklist for Quasi-Experimental Studies and judged as low risk of bias. It was therefore not represented in the traffic-light plot.

Risk of bias was assessed using the JBI Critical Appraisal Checklists. Figure 5 presents the traffic-light plot of the included studies, showing that most studies were rated as low to moderate risk across domains, although several presented concerns regarding confounding factors and sampling strategies. Overall, 8 studies were judged as low risk, 41 as moderate, and 4 as high risk of bias.

## 4. Discussion

### 4.1. Assessment Tools Used to Measure Smartphone Addiction

The primary aim of this review was to identify and synthesise the validated instruments used to assess problematic smartphone use among nursing students. Across the 53 studies included, a total of over ten different tools were identified, reflecting substantial methodological heterogeneity in how this construct is operationalised. The Smartphone Addiction Scale–Short Version (SAS-SV) [65] emerged as the most commonly used instrument, applied in 15 studies, which informed our decision to use it as the basis for the meta-analysis. Other tools included the Nomophobia Questionnaire (NMP-Q) [5], the full version of the Smartphone Addiction Scale (SAS) [66], the Digital Addiction Scale (DAS) [67], and the Smartphone Addiction Inventory (SPAI) [68], among others.

This diversity of tools underscores a lack of standardisation in the field. Despite some shared conceptual foundations, these instruments differ in structure, scoring systems, cut-off thresholds, and psychometric validation. Even among studies using the SAS-SV, there was variation in terms of the language version employed and whether the instrument had undergone local cultural adaptation or revalidation. Such variability not only limits direct comparability across studies but also introduces potential measurement bias that may contribute to the heterogeneity observed in the meta-analytic synthesis.

Given the widespread use of the SAS-SV and its relative brevity and simplicity, it presents a pragmatic option for standardised assessment. However, its dominance in the literature should not obscure the fact that the field currently lacks a universally adopted measurement framework. Achieving greater consistency in assessment practices, including clear reporting of instrument properties and validation procedures, is essential for building a more robust and comparable evidence base.

### 4.2. Meta-Analytic Findings and Methodological Considerations

The meta-analysis focused on studies that employed the Smartphone Addiction Scale–Short Version (SAS-SV), which allowed for a more psychometrically homogeneous synthesis. Eleven studies, comprising a pooled sample of 5586 nursing students, were eligible for inclusion based on complete statistical reporting. This interpretation is consistent with the exploratory scatterplots of SAS-SV scores by female proportion and mean age (Figure 4), which, although inconclusive, suggest that demographic composition may contribute to the extreme heterogeneity observed.

Beyond the quantitative findings, the pooled mean SAS-SV score of 29.5 requires contextualisation against the established cut-off values of 31 for males and 33 for females reported in the original validation studies [65]. This suggests that, on average, nursing students fall just below the threshold for clinical risk, but many individuals likely surpass it, highlighting the vulnerability of this group to problematic smartphone use.

The extremely high heterogeneity (I^2^ = 97.9%) can plausibly be explained by methodological and sample-related differences. Most studies were conducted in Turkey, but variability was also evident across cultural contexts, validated language versions of the SAS-SV, and recruitment strategies (census, quota, convenience). Demographic moderators such as sex distribution (most samples > 70% female), mean age (ranging from 19 to 22 years), and year of data collection may have contributed further dispersion (Figure 5). Although formal meta-regression was not possible, this narrative exploration underscores the need for harmonised reporting of core descriptors in future research.

Importantly, the risk of bias assessment revealed that most studies were of moderate quality, primarily due to insufficient adjustment for confounding factors and limitations in sampling representativeness. These methodological weaknesses likely aggravated the observed heterogeneity, limiting the certainty of pooled estimates.

Mechanistically, the associations observed may reflect bidirectional processes. Excessive smartphone use can impair sleep [7,20,33,46,62], increase stress [20,57], and foster maladaptive coping, while pre-existing stress, low self-esteem, or depressive symptoms may predispose students to problematic use as a self-regulatory strategy. Recognising this bidirectionality is critical for designing effective interventions.

For nursing education, the implications are concrete. Digital self-regulation training could be embedded into curricula, focusing on managing screen time, mitigating distraction during clinical placements, and fostering healthier digital habits. Brief orientation sessions, reflective exercises, and structured guidance from faculty could provide practical strategies to balance the educational utility of smartphones with the risks of overuse.

### 4.3. Psychosocial, Academic, and Clinical Correlates of Smartphone Addiction

Beyond measurement issues, this review identified a consistent pattern of associations between problematic smartphone use and a range of psychosocial and academic variables. Across studies, higher levels of smartphone addiction were regularly linked with elevated stress [37,57,70], anxiety [20,35,59], depressive symptoms [9,28,41], and poorer sleep quality [1,46,62,63]. These findings align with prior literature suggesting that excessive smartphone use may disrupt psychological wellbeing and exacerbate emotional dysregulation.

Additionally, several studies reported negative associations with self-esteem [9,41], increased impulsivity [35], and heightened emotional exhaustion [37,57]. While most of the evidence is cross-sectional and thus limits causal interpretation, the directionality and consistency of these associations across diverse samples and instruments lend weight to their potential relevance.

From an academic standpoint, smartphone addiction was commonly associated with reduced academic performance [1,34], lower motivation [29], and greater levels of procrastination [59]. Some studies also linked excessive use with impaired attention, poor concentration, and dysfunctional decision-making styles. Although the mechanisms remain speculative, it is plausible that persistent digital distraction undermines cognitive engagement and study effectiveness, particularly in high-demand programmes such as nursing.

The findings also raise concerns regarding clinical competencies. Several studies reported correlations between higher addiction scores and diminished communication skills [17,21], lower empathy [37,45], and problematic behaviours such as cyberloafing during placements [56,61]. Moreover, difficulties in clinical decision-making were associated with greater smartphone use in some cohorts [50,56]. These observations highlight the potential impact on professional behaviour and patient care, suggesting that digital overuse may interfere not only with academic success but also with the development of essential clinical attributes.

Taken together, these correlational findings suggest that smartphone addiction is not an isolated behavioural issue but rather intersects meaningfully with multiple dimensions of nursing students’ academic, emotional, and professional functioning. Although causality cannot be assumed, the breadth of associations across studies warrants attention from educators and programme designers.

### 4.4. Limitations of the Evidence Base and Review Process

Several limitations must be acknowledged when interpreting the findings of this review. First, most included studies employed cross-sectional designs and relied exclusively on self-report instruments, which raises concerns about both causal inference and common method bias. The absence of longitudinal or experimental data limits our understanding of the directionality of the observed associations and restricts any interpretation of smartphone addiction as a determinant, rather than a correlate, of psychosocial or academic outcomes.

Second, the sampling methods used across studies were predominantly non-probabilistic, often involving single-institution cohorts, which constrains the external validity of the findings. Many studies also failed to report key demographic variables such as academic year, sex distribution, or year of data collection, which hindered the possibility of conducting moderator analyses or subgroup comparisons. Such omissions reduce the transparency and reproducibility of the evidence base.

Third, there was substantial measurement heterogeneity, not only in the choice of instruments but also in their linguistic versions and levels of psychometric validation. Even among studies using the SAS-SV, differences in translation, cultural adaptation, and scoring conventions may have introduced additional variance. Moreover, several studies failed to specify cut-off scores or interpretive thresholds, limiting their practical applicability.

Regarding the review process itself, four SAS-SV studies were excluded from the meta-analysis [15,17,20,62] due to incomplete or stratified reporting but were retained for narrative synthesis. While standardised conversion rules were applied to harmonise per-item scores into total scores, this may have introduced minor imprecision. Additionally, no formal inter-rater reliability statistics were computed during screening or risk-of-bias assessment, although consensus procedures were followed.

Finally, we did not apply the GRADE approach to assess the overall certainty of the evidence, as this framework is primarily designed for intervention studies and is not routinely recommended for continuous outcomes derived from observational cross-sectional data. Nonetheless, the absence of a formal grading system may limit readers’ ability to gauge the confidence warranted in the pooled estimates.

These limitations do not invalidate the findings, but they do call for greater methodological rigour in future research. Addressing these issues will be essential for improving both the reliability of pooled estimates and the interpretability of their educational and clinical implications.

Although conversions were performed using established formulas and documented in Supplementary Table S1, future studies should report complete summary statistics to minimise the need for such transformations and to facilitate more precise moderator analyses.

### 4.5. Implications for Practice and Future Research

The findings of this review have meaningful implications for both educational and clinical contexts within nursing education. The consistent associations observed between problematic smartphone use and a range of academic, psychological, and professional outcomes highlight the need for proactive institutional strategies. Nursing programmes should consider integrating digital self-regulation training, promoting awareness of the risks associated with excessive smartphone use, and embedding distraction management techniques within the curriculum. Such content may be especially relevant during clinical placements, where professional standards and patient safety must be balanced with students’ need for access to digital resources.

Where institutions contemplate implementing smartphone restrictions during clinical training, policies should be evidence-informed, clearly communicated, and sensitive to the dual role of smartphones as both learning tools and potential sources of distraction. Blanket prohibitions may be counterproductive if they do not address underlying behavioural habits or offer viable alternatives for accessing essential information.

From a research perspective, this review underscores the urgent need for longitudinal and interventional studies to move beyond correlational designs and explore the causal dynamics of smartphone use in nursing education. Future investigations should aim for greater methodological consistency, including the adoption of standardised descriptors (e.g., academic year, sex distribution), consistent reporting of instrument properties, and transparent sampling procedures. Cross-cultural validation of commonly used tools like the SAS-SV is necessary to ensure their applicability across diverse educational settings.

Furthermore, researchers are encouraged to pre-register protocols, use common data elements where possible, and facilitate data sharing to enable more robust meta-analyses. Attention to statistical reporting quality, especially in terms of means, standard deviations, and subgroup stratification, would also improve the reliability and interpretability of future syntheses.

In sum, addressing smartphone addiction in nursing students will require coordinated efforts across educational policy, pedagogy, and research methodology. This review highlights both the scope of the problem and the foundational steps needed to build a more cohesive and actionable evidence base.

## 5. Conclusions

This systematic review and meta-analysis identified the Smartphone Addiction Scale–Short Version (SAS-SV) as the most frequently applied instrument to evaluate problematic smartphone use in nursing students. The pooled mean SAS-SV score indicated a moderate level of addiction risk, though results showed very high heterogeneity and frequent methodological limitations.

These findings must be interpreted with caution, given that most studies were cross-sectional and often lacked adjustment for confounding factors. Nevertheless, the consistent associations with psychosocial and academic outcomes reinforce that excessive smartphone use is a genuine concern in nursing education.

For practice and policy, our results underline the need for nursing schools to provide structured guidance on digital self-regulation, integrate training on distraction management into curricula and clinical placements, and promote institutional policies that balance smartphone utility with patient care quality. At the regulatory level, standardised assessment tools—particularly the SAS-SV, given its widespread use—should be prioritised and cross-culturally validated to enable more robust evidence and international comparability.

Future research should move beyond cross-sectional designs and develop longitudinal and interventional studies to clarify causal pathways and to evaluate targeted digital literacy and wellbeing programmes within nursing education.

## Figures and Tables

**Table 1:** Eligibility criteria for study inclusion in the systematic review.

Criterion	Inclusion	Exclusion
Population	Undergraduate/pre-licensure nursing students	Other health sciences students (unless nursing subgroup data extractable); midwifery-only cohorts; patient populations
Study design	Quantitative, analytical cross-sectional studies; quasi-experimental (baseline only)	Qualitative studies; literature reviews; editorials; opinion pieces; case reports
Outcomes	Smartphone addiction/problematic use assessed with validated instruments; quantitative scores or prevalence estimates reported	Non-validated tools; lack of quantitative data
Language	English or Spanish	Other languages
Time frame	Jan 2014–May 2024	Outside the specified range
Meta-analysis inclusion	SAS-SV studies with complete statistical data (*n*, mean, SD or convertible equivalents)	Stratified data without subgroup pooling; approximated pooled means (except for sensitivity analyses)

**Table 2:** Summary of the studies included in the systematic review (N = 53).

Author (Year), Country	Sample Size (*n*)	Main Instrument(s)	Key Findings
Akturk & Budak (2019), Turkey [16]	1049	SAS-SV, MSPSS	Smartphone addiction was negatively correlated with perceived social support, including all subscales: family, friends, and significant others
Alsayed et al. (2020), Saudi Arabia [3]	135	Expert-validated ad hoc questionnaire	High academic use of smartphones was reported, but no significant associations were found with academic performance or health-related outcomes
Ayar & Gürkan (2021), Turkey [17]	587	SAS-SV, Phubbing Scale, Communication Skills	Communication skills were negatively associated with both smartphone addiction and phubbing behaviours, with both variables jointly explaining 60% of the variance
Bajamal et al. (2023), Saudi Arabia [13]	133	SAS-SV	No significant correlation was found between smartphone overuse and academic performance; most students reported frequent use for study purposes
Barzegari et al. (2023), Iran [28]	281	SPAI-PV, PHQ-9	Significant positive correlation found between smartphone addiction and depression
Bayir & Topbas (2023), Turkey [23]	82	Technology Addiction Scale	Moderate addiction levels in both groups; the 10-week training had no significant effect on addiction scores
Berdida & Grande (2023), Philippines [29]	835	MSLQ, MTUAS, NMP-Q	Nomophobia was positively associated with social media use and negatively with motivation and attention, which in turn mediated its negative effect on academic performance
Bilgic et al. (2023), Turkey [10]	541	SAS-SV, PRS	Negative correlation between addiction and peer relationships
Catiker et al. (2021), Turkey [30]	97	SAS, FoMO, Care-Q	Association with FoMO and caring behaviors in accessibility and comfort
Çelik İnce (2021), Turkey [31]	607	NMP-Q, Self-Esteem Rating Scale-Short Form	Moderate nomophobia levels found; no significant correlation with self-esteem or obesity
Celikkalp et al. (2020), Turkey [21]	292	SAS-SV, Communication Skills Scale	Association with daily smartphone usage time and academic achievement
Cerit et al. (2018), Turkey [32]	214	SAS, CSS	Smartphone addiction is negatively associated with communication skills; significant effects on self-expression and non-verbal communication identified via regression analysis
Chen et al. (2022), China [33]	1827	BPS, TIPI-C, SRF-S, FPS, SQAPMPU	Problematic mobile phone use significantly predicted higher levels of bedtime procrastination, along with self-regulatory fatigue. Personality traits such as conscientiousness and neuroticism were protective, whereas extraversion was a risk factor
Cho & Lee (2016), South Korea [2]	312	Expert-validated questionnaire (use and distraction)	46.2% used smartphones during clinical practice; 24.7% felt distracted
Çobanoğlu et al. (2021), Turkey [15]	215	SAS-SV, DAS, NMP-Q	Significant positive correlation between addiction and nomophobia
Dayapoğlu et al. (2016), Turkey [34]	353	PMPUS, SWLS, UCLA Loneliness Scale	Problematic use negatively correlated with life satisfaction and GPA, positively with loneliness
Demiralp et al. (2021), Turkey [12]	419	SAS-SV, Daily Goals Scale	Smartphone use affects daily goal setting
El-Ashry et al. (2024), Egypt [35]	1626	NMP-Q, Impulsive Sensation Seeking Scale	Moderate-to-high nomophobia levels associated with impulsivity
Eskin Bacaksiz et al. (2022), Turkey [36]	802	NMP-Q, Fırat Netlessphobia Scale, FoMO Scale	Moderate correlation between nomophobia and netlessphobia; FoMO also correlates
Ghosh et al. (2021), India [7]	91	SAS, PSQI	Smartphone addiction was significantly associated with age, and poor sleep quality was common, though no significant association was found between SAS and PSQI
Gutiérrez-Puertas et al. (2020), Spain [37]	135	WANIS, PSS, ICCI, JSE	Nomophobia levels differed significantly between Spanish and Portuguese students, with Portuguese students showing higher mean scores
Gutiérrez-Puertas et al. (2019), Spain and Portugal [38]	258	NMP-Q	Nomophobia levels differed significantly between Spanish and Portuguese students, with Portuguese students showing higher mean scores
Han et al. (2022), South Korea [39]	197	SAS (proneness), ICQ, Media Multitasking Motivation, Phubbing Scale	Phubbing was positively associated with smartphone addiction and media multitasking, and negatively associated with interpersonal competence. Predictors of phubbing included lower interpersonal competence
İlter & Ovayolu (2022), Turkey [40]	202	SMAS-AF, TAS-20	Significant correlation between addiction and alexithymia; 46% of students were fully alexithymic
Jose et al. (2024), India [41]	402	MPPUS-10, PHQ-9, ISI, SWLS, Rosenberg Self-Esteem	Severe problematic mobile phone use prevalence was 39%. It showed positive correlations with age, depression, and insomnia, and strong negative correlations with satisfaction with life and self-esteem.
Kalal et al. (2023), India [1]	160	SAS-SV, PSQI	Moderate addiction associated with poor sleep and lower academic performance
Kargın et al. (2020), Turkey [42]	511	IAT, FoMO	Positive correlation found between internet addiction and fear of missing out; 3.8% were pathological users, 29.1% at risk. Internet addiction was higher in males
Khatgaonkar et al. (2020), India [43]	100	Ad hoc questionnaire	70% reported being addicted; 77% perceived negative effects on academic performance; 85% reported psychosocial/physical problems. Descriptive report; no detailed statistical analysis
Lee et al. (2018), South Korea [44]	324	SAI, MSPSS, K-ICQ	Positive effects of cyberspace-oriented relationships and perceived social support on interpersonal competence. Other smartphone addiction subscales showed no significant association with interpersonal competence
Lee et al. (2022) Malaysia [45]	345	DAS, IGDS9-SF, TEQ	Increased digital use and gaming correlated with lower empathy and higher callousness; digital-related emotional states also predicted lower empathy and higher callousness.
Lobo et al. (2022), Brazil [46]	298	SPAI, PSQI, AUDIT	Prevalence of smartphone addiction was 47.7%; addiction correlated with poor sleep quality, alcohol use, and daytime dysfunction
Machado et al. (2023), India [47]	270	SAS, Semi-structured questionnaire	Most students were classified as moderately addicted; no significant associations were found with age, gender, or academic level. Reported symptoms included headaches, eye strain, and sleep disturbances.
Mancheri et al. (2023) [48]	234	IAT, CPAS	Highter cell phone addiction in younger and single students; higher internet addiction among dormitory residents; no association with GPA
Marletta et al. (2021), Italy [49]	244	NMP-Q, clinical questionnaire	Nomophobia positively correlated with time spent using the smartphone; significant differences were found in usage during internships
Márquez-Hernández et al. (2020), Spain [50]	124	NMP-Q, MPPUS, MDMQ	Nomophobia was positively correlated with procrastination, hypervigilant and buck-passing decision-making styles
Mersal et al. (2024), Saudi Arabia [51]	227	SAS-SV, NMQ	Smartphone addiction was significantly associated with musculoskeletal pain in the neck, back, and wrists
Mersin et al. (2020), Turkey [52]	272	Toronto Alexithymia Scale	As time spent on social media increases, alexithymia scores and difficulty in recognizing feelings also increase
Mohamed & Mostafa (2020), Egypt [9]	320	SAS, Hamilton Depression, Self-Esteem Inventory	Positive correlation with depression and negative correlation with self-esteem
Oh & Oh (2017), South Korea [53]	329	NISA Smartphone Addiction Proneness Scale	Negative correlations between smartphone addiction and self-esteem and showed pure correlations between self-esteem and empathy
Ozdil et al. (2022), Turkey [54]	259	SAS-SV, Numeric Rating Scale (NRS)	Association smartphone addiction with higher severity of headache, ear pain, shoulder pain and lower back pain
Özer et al. (2023), Turkey [55]	463	IAS, CSS, DERS-16	Internet addiction was negatively correlated with communication skills and positively with emotional regulation difficulties
Savci et al. (2021), Turkey [56]	379	SAS-SV, CLAS, CDMNS	Smartphone addiction positively correlated with cyberloafing and negatively correlated with clinical decision-making
Sok et al. (2019), South Korea [57]	139	Self-Control Scale, Daily Life Stress Scale, GICC	Nursing students in the smartphone addiction risk group had significantly lower self-control and higher daily life stress than the general group; no significant difference in communication skills
Sönmez et al. (2020), Turkey [58]	682	SAS-SV, UCLA Loneliness Scale	Positive correlation between smartphone addiction and loneliness
Tárrega-Piquer et al. (2023), Spain [59]	308	NMP-Q, SAQ, APS-SF	Nomophobia affected 19.5%; NMP-Q was higher with more daily use and in-class checking, inversely related to self-reported grades, not related to procrastination
Tastan et al. (2021), Turkey [20]	333	SAS-SV, Interaction Anxiousness Scale	Smartphone addiction correlated with higher social anxiety in interaction situations
Turan et al. (2020), Turkey [60]	160	IAS, UCLA, SWLS	Internet addiction was at a moderate level, no significant correlation between internet addiction, loneliness, and life satisfaction. A positive correlation was found between loneliness and life satisfaction.
Turan et al. (2021), Turkey [61]	518	SMAS, CLS	Moderate positive correlation between social media addiction and cyberloafing
Uzuncakmak et al. (2022), Turkey [62]	771	SAS-SV, PSQI, Epworth Sleepiness Scale	High smartphone addiction related to poorer sleep quality and more daytime sleepiness
Yaman Aktaş et al. (2022), Turkey [63]	429	DAS, Level 2-Sleep Disturbance	Positive correlation between digital addiction and sleep disorders
Yatmaz et al. (2022), Turkey [8]	310	SAS, Life Goals Scale	Significant relationship between mobile addiction and reduced life goal clarity
Zhao (2022), China [64]	568	FFMQ, LOT-R, Loneliness Scale, SDL Scale	Mindfulness and optimism positively associated; loneliness negatively associated with SDL
Zhou et al. (2022), China [27]	1445	SAS-SV, IPASN, ASES, ABS	Positive correlation between smartphone addiction and academic burnout

Notes (instruments/acronyms): ABS = Academic Burnout Scale; ASES = Academic Self-Efficacy Scale; APS-SF = Academic Procrastination Scale–Short Form; AUDIT = Alcohol Use Disorders Identification Test; BPS = Bedtime Procrastination Scale; Care-Q = Caring Assessment Questionnaire; CDMNS = Clinical Decision-Making in Nursing Scale; CLAS = Cyberloafing Academic Scale; CLS = Cyberloafing Scale; CSS = Communication Skills Scale; CSAS = Communication Skills Assessment Scale; DAS = Digital Addiction Scale; DERS-16 = Difficulties in Emotion Regulation Scale (16 items); DGS = Daily Goals Scale; ESS = Epworth Sleepiness Scale; FFMQ = Five Facet Mindfulness Questionnaire; FoMO = Fear of Missing Out Scale; FPS = Future Perspective Scale; GICC = Global Interpersonal Communication Competence Scale; IAS = Internet Addiction Scale (note: context-dependent; in some studies used for Internet Addiction, in others for Interaction Anxiousness Scale); ICCI = Interpersonal Communication Competence Inventory; ICQ/K-ICQ = Interpersonal Competence Questionnaire/Korean version; ImpSS = Impulsive Sensation Seeking Scale; IGDS9-SF = Internet Gaming Disorder Scale–Short Form; IPASN = Inventory of Professional Attitude for Student Nurses; IRI = Interpersonal Reactivity Index; ISI = Insomnia Severity Index; JSE = Jefferson Scale of Empathy; LOT-R = Life Orientation Test–Revised; MDMQ = Melbourne Decision Making Questionnaire; MMM = Media Multitasking Motivation; MPPUS/MPPUS-10 = Mobile Phone Problem Use Scale/10-item short form; MSLQ = Motivated Strategies for Learning Questionnaire; MSPSS = Multidimensional Scale of Perceived Social Support; MTUAS = Media and Technology Usage and Attitudes Scale; NISA/SAP-NISA = Smartphone Addiction Proneness Scale (National Information Society Agency, Korea); NMP-Q = Nomophobia Questionnaire; NMQ = Nordic Musculoskeletal Questionnaire; NRS = Numeric Rating Scale (for pain); PB = Phubbing Behavior subscale (from the Phubbing Scale); PHQ-9 = Patient Health Questionnaire-9 (depression); PMPUS = Problematic Mobile Phone Use Scale; PRS = Peer Relations Scale; PSQI = Pittsburgh Sleep Quality Index; SAI/SPAI/SPAI-PV = Smartphone Addiction Inventory/Persian Version; SAQ/SAQ-A30 = Social Anxiety Questionnaire for Adults (30 items); SAS = Smartphone Addiction Scale (33 items); SAS-SV = Smartphone Addiction Scale–Short Version (10 items); SASp = Smartphone Addiction Scale–Proneness version; SDL Scale = Self-Directed Learning Scale; Self-Esteem Inventory/Rosenberg Self-Esteem = Rosenberg Self-Esteem Scale; SMAS/SMAS-AF = Social Media Addiction Scale/Adult Form; SQAPMPU = Short Questionnaire for Assessing Problematic Mobile Phone Use; SRF-S = Self-Regulatory Fatigue Scale–Short; SWLS = Satisfaction With Life Scale; TAS-20 = Toronto Alexithymia Scale–20; TEQ = Toronto Empathy Questionnaire; TIPI-C = Ten-Item Personality Inventory–Chinese version; UCLA = UCLA Loneliness Scale; WANIS = WhatsApp Addiction and Negative Impact Scale (ad hoc, Gutiérrez-Puertas et al. [37]; Level2SD = Level 2–Sleep Disturbance scale (DSM-5 Self-Rated Level 2 Cross-Cutting Symptom Measure).

**Table 3:** Questionnaires Used in Studies Related to Smartphone Use.

Authors and Year	Questionnaires or Scales Used	Number of Items	Item Format	Scoring Scale	Domains Assessed	Frequency of Use in Included Studies (k)
Kwon et al. (2013) [65]	Smartphone Addiction Scale–Short Version (SAS-SV)	10	Likert scale	1–6	Similar to the SAS, but shorter and easier to administer	15
Yildirim & Correia (2015) [5]	Nomophobia Questionnaire (NMP-Q)	20	Likert scale	1–7	Levels of nomophobia	9
Kwon et al. (2013) [66]	Smartphone Addiction Scale (SAS)	33	Likert scale	1–6	Levels of smartphone addiction	6
Kesici & Tunç (2018) [67]	Digital Addiction Scale (DAS)	19	Likert scale	1–5	Overuse, Non-restraint, Inhibiting the Flow of Life, Emotional State, Dependence	3
Lin et al. (2014) [68]	Smartphone Addiction Inventory (SPAI)	26	Likert scale	1–4	Levels of smartphone addiction	2
Bianchi & Phillips (2005) [69]	Mobile Phone Problematic Use Scale (MPPUS)	27	Likert scale	1–10	Assessing mobile phone addiction and problematic use	2
Billieux et al. (2008) [70]	Problematic Mobile Phone Use Questionnaire (PMPUQ)	30	Likert scale	1–4	Dependency symptoms, dangerous use, negative social/emotional consequences	1
Rosen et al. (2013) [71]	Media and Technology Usage and Attitudes Scale (MTUAS)	60	Likert scale	1–5	Attitudes towards media and technology use	1
Foerster et al. (2015) [72]	Mobile Phone Problematic Use Scale-10 (MPPUS-10)	10	Likert scale	1–10	Levels of problematic mobile phone use	1
Koo (2009) [73]	Cell Phone Addiction Scale (CPAS)	20	Likert scale	1–5	Identification of mobile phone addiction levels	1

**Table 4:** Characteristics of Studies Included in the Meta-analysis Using the Smartphone Addiction Scale–Short Version (SAS-SV).

ID	Author (Year)	Country	N	Mean (SD)	Instrument Version	Sample Demographics	Sampling Strategy	Risk of Bias (JBI)
1	Akturk & Budak [16]	Turkey	1049	28.29 ± 11.92	Turkish validated	62.7% female, age 21.54 ± 2.27	Census, 95.8% response	Moderate
2	Bajamal et al. [13]	Saudi Arabia	133	34.30 ± 8.90	English validated	100% female, age 21.70 ± 1.04	Quota, NR	Low
3	Bilgic et al. [10]	Turkey	541	26.10 ± 11.16	Turkish validated	78.9% female, age 20.17 ± 1.75	Census, 79.2% response	Moderate
4	Celikkalp et al. [21]	Turkey	292	33.32 ± 9.54	Turkish validated	70.9% female, age NR	Census, 69.9% response	Moderate
5	Demiralp et al. [12]	Turkey	419	29.23 ± 10.73	Turkish validated	87.2% female, age 19.75 ± 1.43	Census	Moderate
6	Kalal et al. [1]	India	160	26.44 ± 8.67	English validated	98.1% female, age 21.70 ± 1.55	Census	Moderate
7	Mersal et al. [51]	Saudi Arabia	227	27.60 ± 8.30	Language NR	62.6% female, age 19.33 ± 1.19	Convenience, 53.4% response	Low
8	Ozdil et al. [54]	Turkey	259	25.71 ± 7.49	Turkish validated	80.7% female, age 20.29 ± 1.60	Stratified, 61.4% response	Moderate
9	Savci et al. [56]	Turkey	379	29.22 ± 9.89	Turkish validated	76.0% female, age 20.36 ± 1.17	Census, 90.2% response	Low
10	Sönmez et al. [58]	Turkey	682	31.40 ± 10.17	Turkish validated	74.5% female, age 20.76 ± 1.72	Census, 72.1% response	Moderate
11	Zhou et al. [27]	China	1445	32.92 ± 8.05	Chinese validated	NR, age 19.65 ± 1.35	Convenience, 96.0% response	Low

**Table 5:** Summary of meta-analysis results.

Statistic	Estimate (95% CI)	*p*-Value	Notes
Pooled mean SAS-SV	29.50 (27.70–31.29)	<0.001	Random-effects (REML)
Prediction interval	23.36–35.64	–	Indicates expected range in future studies
Heterogeneity (I^2^)	97.90%	<0.001	Very high
Between-study variance (τ^2^)	8.97	–	
Cochran’s Q	485.20 (df = 10)	<0.001	
Egger’s test	*p* = 0.982	–	No evidence of small-study effects
Kendall’s τ	−0.018 (*p* = 0.94)	–	Consistent with Egger

## Data Availability

The datasets used and/or analysed during the current study are available from the corresponding author upon reasonable request.

## Supplementary Materials

The following supporting information can be downloaded at: https://www.mdpi.com/article/10.3390/healthcare13202639/s1, Table S1: Data conversions applied in the SAS-SV meta-analysis; Table S2: Key characteristics of the studies included in the systematic review (N = 53); Table S3: Critical appraisal of the included studies using the JBI Checklist for Analytical Cross-Sectional Studies.

## Abbreviations

The following abbreviations are used in this manuscript:

Abbreviation	Definition
CINAHL	Cumulative Index to Nursing and Allied Health Literature
CI	Confidence Interval
FoMO	Fear of Missing Out
I^2^	Higgins’ heterogeneity index
JBI	Joanna Briggs Institute
NMP-Q	Nomophobia Questionnaire
PSQI	Pittsburgh Sleep Quality Index
PRISMA	Preferred Reporting Items for Systematic Reviews and Meta-Analyses
PROSPERO	International Prospective Register of Systematic Reviews
SAS	Smartphone Addiction Scale
SAS-SV	Smartphone Addiction Scale–Short Version
