# Supplementary material for: Assessment Tools and Psychosocial Consequences of Smartphone Addiction in Nursing Students: A Systematic Review and Meta-Analysis
Source: Healthcare (Basel). 2025 Oct 20;13(20):2639. doi: 10.3390/healthcare13202639 (PMC12563757; doi:10.3390/healthcare13202639)
Supplement: Supplementary file 1 [file healthcare-13-02639-s001.zip › Supplementary Table S1.pdf]

Table S1: Data conversions applied in the SAS-SV meta-analysis

Situation in primary study	Conversion applied	Formula used	Example from included studies
Only per-item mean reported (SAS-SV, 1–6 Likert)	Converted to total score	**Total = Mean per item × 10**	*Celikkalp et al. (2020):* 3.33 per item → 33.3 total
Stratified data (e.g., by academic year)	Pooled into single estimate	**Mean pooled = (Σ ni mi)/(Σ ni)**; **SD pooled = √{[Σ (ni–1)SDi² + Σ ni(mi – Mpooled)²]/(Σ ni –1)}**	*Tastan et al. (2021):* year 1 (n=120, m=30.1, SD=8.9) + year 2 (n=130, m=29.4, SD=9.1) → pooled mean 29.7, SD 9.0
Only SE reported	Converted to SD	**SD = SE × √n**	*Han et al. (2022):* SE=0.42, n=300 → SD=0.42×√300=7.27
Only 95% CI reported	Converted to SD	**SD = √n × (Upper–Lower)/3.92**	*Zhou et al. (2022):* mean=28.5, CI95% 27.8–29.2, n=250 → SD=√250×(29.2–27.8)/3.92=8.89
