# Supplementary material for: Assessment Tools and Psychosocial Consequences of Smartphone Addiction in Nursing Students: A Systematic Review and Meta-Analysis
Source: Healthcare (Basel). 2025 Oct 20;13(20):2639. doi: 10.3390/healthcare13202639 (PMC12563757; doi:10.3390/healthcare13202639)
Supplement: Supplementary file 1 [file healthcare-13-02639-s001.zip › Supplementary Table S2.pdf]

Table S2 Key characteristics of the studies included in the systematic review (N = 53).

Author (Year), Country	Study Design	Sample (n, % female)	Mean Age ± SD	Instrument(s) Used	Main Variable	Mean score of instrument(s) used	Main Findings
Akturk & Budak (2019), Turkey[16]	TC	1049, 62.7%	21.54 ± 2.27	SAS-SV, MSPSS	Smartphone addiction and social support	SAS-SV= 28.29 ± 11.92*; MSPSS: 57.6 ± 15.5	Smartphone addiction was negatively correlated with perceived social support (r = –.111, p < .001), including all subscales: family (r = –.096), friends (r = –.470), and significant others (r = –.115).
Alsayed et al. (2020), Saudi Arabia[3]	TD	135, 100%	21.84 ± 1.25	Expert-validated ad hoc questionnaire	Academic use of smartphones	NR	High academic use of smartphones was reported, but no significant associations were found with academic performance or health-related outcomes.
Ayar & Gürkan (2021), Turkey[17]	TC	587, 69.3%	20.47 ± 1.91	SAS-SV, Phubbing Scale, Communication Skills	Smartphone addiction, phubbing, and communication skills	NR	Communication skills were negatively associated with both smartphone addiction and phubbing behaviours (β = –0.314 and –0.503, respectively; p < .001), with both variables jointly explaining 60% of the variance.
Bajamal et al. (2023), Saudi Arabia[13]	TD	133, 100%	21.7 ± 1.04	SAS-SV	Smartphone addiction and academic performance	SAS-SV= 34.3 ± 8.90	No significant correlation was found between smartphone overuse and academic performance (r = –0.103, p > .05); most students reported frequent use for study purposes.
Barzegari et al. (2023), Iran[23]	TC	281, 55.2%	20.9 ± 2.57	SPAI-PV, PHQ-9	Smartphone addiction and depression	SPAI-PV = 55.86 ± 14.17	Significant positive correlation found between smartphone addiction and depression (r = 0.47, p < 0.001).
Bayir & Topbas (2023), Turkey[22]	ED	82, 76%	GE: 19.37 ± 1.71<br>GC: 22.32 ± 2.81.	Technology Addiction Scale	Technological addiction in health sciences students	GE: 52.69 ± 17.22 (pre) → 51.31 ± 14.93 (post); GC: 54.03 ± 18.96 → 53.39 ± 20.16	Moderate addiction levels in both groups; the 10-week training had no significant effect on addiction scores (p > 0.05).

Berdida & Grande (2023), Philippines[24]	TC	835, 79.7%	20.43 ± 6.85	MSLQ, MTUAS, NMP-Q	Nomophobia, social media use, motivation, and attention	NMP-Q: 83.32 ±23.30*	Nomophobia was positively associated with social media use and negatively with motivation and attention, which in turn mediated its negative effect on academic performance ($p < 0.05$).
Bilgic et al. (2023), Turkey[10]	TC	541, 82.2%	20.17 ± 1.75	SAS-SV, PRS	Smartphone addiction and peer relationships	SAS-SV: 26.10 ± 11.16; PRS: 67.35 ± 12.43	Negative correlation between addiction and peer relationships ($r = -0.170$, $p < 0.01$)
Catiker et al. (2021), Turkey[25]	TC	97, 82.5%	21.39 ± 0.68	SAS, FoMO, Care-Q	Smartphone addiction, FoMO, and caring behaviours	SAS: 83.30 ± 2.21; FoMO: 29.64 ± 8.77; Care-Q: 4.63 ± 0.80	Association with FoMO and caring behaviors in accessibility and comfort ($p < 0.05$)
Çelik İnce (2021), Turkey[26]	TC	607, 75.5%	20.53 ± 1.83	NMP-Q, Self-Esteem Rating Scale-Short Form	Nomophobia, self-esteem, and obesity	NMP-Q: 74.43 ±23.68	Moderate nomophobia levels found; no significant correlation with self-esteem ($r = –0.067$, $p = .097$) or obesity ($r = 0.043$, $p = .295$).
Celikkalp et al. (2020), Turkey[27]	TC	292, 70.9%	NR	SAS-SV, Communication Skills Scale	Smartphone addiction and communication skills	SAS-SV: 33.32 ± 9.54	Association with daily smartphone usage time and academic achievement ($p < 0.05$)
Cerit et al. (2018), Turkey[28]	TC	214, 80.4%	20.39 ± 1.45	SAS, CSS	Smartphone addiction and communication	SAS: 86.43 ± 29.66; CSAS: 97.99 ± 13.17	Smartphone addiction negatively associated with communication skills ($r = –.149$, $p = .029$); significant effects on self-expression and non-verbal communication identified via regression analysis.

Chen et al. (2022), China[65]	TD	1827, 75.5%	19.07 ± 1.09	BPS, TIPI-C, SRF-S, FPS, SQAPMPU	Bedtime procrastination	BPS: 25.11 ± 6.88	Problematic mobile phone use significantly predicted higher levels of bedtime procrastination (β = 0.078, p < .001), along with self-regulatory fatigue (β = 0.135, p < .001). Personality traits such as conscientiousness (β = –0.284, p = .003) and neuroticism (β = –0.203, p = .031) were protective, while extroversion (β = 0.225, p = .001) was a risk factor. Future time perspective was negatively associated (β = –0.141, p < .001). Together, predictors explained 21.7% of variance in bedtime procrastination.
Cho & Lee (2016), South Korea[2]	TD	312, 89.1%	21.7 ± 2.59	Expert-validated questionnaire (use and distraction)	Clinical smartphone use and policy perception	NR	46.2% used smartphones during clinical practice; 24.7% felt distracted
Çobanoğlu et al. (2021), Turkey[15]	TC	215, 76.3%	20.91 ± 2.06	SAS-SV, DAS, NMP-Q	Smartphone addiction and nomophobia	NR	Significant positive correlation between addiction and nomophobia (r = 0.628, p < 0.01)
Dayapoğlu et al. (2016), Turkey[30]	TD	353, 77.6%	20.65 ± 1.65	PMPUS, SWLS, UCLA Loneliness Scale	Problematic mobile use and well-being	PMPUS: 55.91 ± 18.66; SWLS: 22.05 ± 6.00; UCLA: 39.11 ± 7.00	Problematic use negatively correlated with life satisfaction and GPA, positively with loneliness (p < 0.01).
Demiralp et al. (2021), Turkey[12]	TC	419, 87.2%	19.75 ± 1.43	SAS-SV, Daily Goals Scale	Smartphone addiction and daily goal-setting	SAS-SV: 29.23 ± 10.73; DGS: 49.58 ± 6.0	Smartphone use affects daily goal setting (p < 0.05)
El-Ashry et al. (2024), Egypt[31]	TC	1626, 61.9%	NR	NMP-Q, Impulsive Sensation Seeking Scale	Nomophobia and impulsivity	NMP-Q: 82.32 ± 36.14; ImpSS: 115.36 ± 23.35	Moderate-to-high nomophobia levels associated with impulsivity (p < 0.01)

Eskin Bacaksiz et al. (2022), Turkey[32]	TC	802, 78.9%	20.53 ± 1.77	NMP-Q, Fırat Netlessphobia Scale, FoMO Scale	Nomophobia, netlessfobia, FoMO	NR	Moderate correlation between nomophobia and netlessphobia (r = 0.666); FoMO also correlates (r = 0.430).
Ghosh et al. (2021), India[7]	TC	91, 100%	NR	SAS, PSQI	Smartphone addiction and sleep quality	SAS: 102.85 ± 19.17; PSQI; 10.87 ± 5.49.	Smartphone addiction was significantly associated with age (p = .000031), and poor sleep quality was common (82.42%), though no significant association was found between SAS and PSQI scores (p = 0.36).
Gutiérrez-Puertas et al. (2020), Spain[33]	TC	135, 83.7%	22.9 ± 4.75	WANIS, PSS, ICCI, JSE	Impact of WhatsApp use	WANIS: 32.5 ± 8.1; PSS: 31.17 ± 6.44; ICCI: 53.17 ± 6.86; JSE: 86.64 ± 8.08	Nomophobia levels differed significantly between Spanish and Portuguese students (p < .001), with Portuguese students showing higher mean scores.
Gutiérrez-Puertas et al. (2019), Spain and Portugal[34]	TD	258, 81%	20.78 ± 3.16	NMP-Q	Nomophobia	NMP-Q: 89.43 ± 26.53	Nomophobia levels differed significantly between Spanish and Portuguese students (p < .001), with Portuguese students showing higher mean scores.
Han et al. (2022), South Korea[66]	TC	197, 67.5%	22.69 ± 2.56	SAS(proneness), ICQ, Media Multitasking Motivation, Phubbing Scale	Smartphone addiction, phubbing, interpersonal competence	PB: 26.51±7.77; SASp: 35.66 ± 9.25 ; MMM: 71.51±14.21; ICQ: 112.41±17.43	Phubbing was positively associated with smartphone addiction (r = 0.41, p < .001) and media multitasking (r = 0.16, p < .05), and negatively associated with interpersonal competence (r = -0.51, p < .001). Predictors of phubbing included lower interpersonal competence, higher multitasking motivation, and higher smartphone addiction, explaining 43% of the variance.
İlter & Ovayolu (2022), Turkey[36]	TC	202, 75.2%	NR	SMAS-AF, TAS-20	Social media addiction and alexithymia	SMAS-AF: 64.81 ± 5.80; TAS-20: 71.27 ± 8.62	Significant correlation between addiction and alexithymia (p < 0.05) ; 46% of students met the criteria for alexythimia

Jose et al. (2024), India[37]	TC	402, 91.3%	20.47 ± 1.62	MPPUS-10, PHQ-9, ISI, SWLS, Rosenberg Self-Esteem	Mobile addiction and mental health	MPPUS-10: 42.19 ± 21.13	Severe problematic mobile phone use prevalence was 39%. It showed positive correlations with age, depression, and insomnia (p < 0.05), and strong negative correlations with satisfaction with life and self-esteem (p < 0.05). Depression and satisfaction with life mediated the effect of self-esteem on problematic use.
Kalal et al. (2023), India[1]	TC	160, 98.1%	21.7 ± 1.55	SAS-SV, PSQI	Smartphone addiction and sleep quality	SAS_SV: 26.44 ± 8.67; PSQI: 4.73 ± 3.10	Moderate addiction associated with poor sleep and lower academic performance (p < .001)
Kargın et al. (2020), Turkey[38]	TD	511, 72.6%	NR	IAT, FoMO	Internet addiction and FoMO	IAS: 42.17 ± 19.09; FoMOs: 23.27 ± 8.08	Positive correlation found between internet addiction and fear of missing out (FoMO) (r = .332, p < .001); 3.8% were pathological users, 29.1% at risk. Internet addiction was higher in males and in the 21–25 age group.
Khatgaonkar et al. (2020), India[39]	TD	100, NR	NR	Ad hoc questionnaire	Problematic mobile use and symptoms	NR	70% reported being addicted; 77% perceived negative effects on academic performance; 85% reported psychosocial/physical problems (headache, eye pain, thumb pain, backache). Descriptive report; no detailed statistical analysis
Lee et al. (2018), South Korea[40]	TD	324, 91.4%	NR	SAI, MSPSS, K-ICQ	Smartphone addiction, social support, interpersonal competence	SAI: 47.83 ± 11.95; MSPSS: 67.79 ± 10.12; K-ICQ: 97.73 ± 16.38	Positive effects of cyberspace-oriented relationships (β = 1.360, p = .004) and perceived social support (β = 0.555, p < .001) on interpersonal competence. Other smartphone addiction subscales showed no significant association with interpersonal competence.
Lee et al. (2022) Malaysia[41]	TC	345, 86,7%	20.16 ± 1.15	DAS, IGDS9-SF, TEQ	Digital Addiction, Internet Gaming and Empathy	DAS = 2.75 ± 0.55; IGDS9-SF = 16.17 ± 6.17; TEQ = 49.20 ± 6.87	Increased digital use and gaming correlated with lower empathy and higher callousness (β = −0.192, β = 0.131); digital-related emotional states also predicted lower empathy (β = −0.111) and higher callousness (β = 0.181). However, digital dependence was positively associated with empathy (β = 0.172). Internet gaming addiction predicted higher callousness (β = 0.265).

Lobo et al. (2022), Brazil[42]	TC	298, 80.2%	22 ± 3	SPAI, PSQI, AUDIT	Smartphone addiction, alcohol use and sleep quality	NR	Prevalence of smartphone addiction was 47.7%; addiction correlated with poor sleep quality, alcohol use, and daytime dysfunction ($p < 0.05$)
Machado et al. (2023), India[43]	TC	270, 91.1%	20.89±3.09	SAS, Semi-structured questionnaire	Excessive smartphone use and academic performance	SAS: 91.34 ± 27.00*	Most students were classified as moderately addicted; no significant associations were found with age, gender, or academic level ($p > 0.05$). Reported symptoms included headaches (40.2%), eye strain (30.6%), and sleep disturbances (22%). Smartphone use did not significantly impact study habits, with 98.1% reporting good or average habits .
Mancheri et al. (2023)[44]	TD	234, NR	NR	IAT, CPAS	Internet and mobile addiction	IAT: 45.15 ± 12.44; CPAS: 51.95 ± 14.74	Highter cell-phone addiction in younger ($p=0.011$) and single students ($p=0.038$); higher internet addiction among dormitory residents ($p=0.011$); no association with GPA ($p>0.05$).
Marletta et al. (2021), Italy[45]	TD	244, NR	NR	NMP-Q, clinical questionnaire	Nomophobia and clinical use	NR	Nomophobia positively correlated with time spent using the smartphone ($Rs = .287$, $p < .01$); significant differences were found in usage during internships ($\chi^2 = 93.755$, $p < 0.01$).
Márquez-Hernández et al. (2020), Spain[46]	TC	124, 79%	20.92 ± 5.34	NMP-Q, MPPUS, MDMQ	Nomophobia and decision-making	NR	Nomophobia was positively correlated with procrastination ($rs = 0.307$), hypervigilant ($rs = 0.284$) and buck-passing ($rs = 0.263$) decision-making styles ($p < 0.05$).
Mersal et al. (2024), Saudi Arabia[47]	TC	227, 62.6%	19.33 ± 1.19	SAS-SV, NMQ	Smartphone addiction and musculoskeletal health	27.60 ± 8.30	Smartphone addiction was significantly associated with musculoskeletal pain in the neck, back, and wrists ($p < 0.05$).
Mersin et al. (2020), Turkey[48]	TC	272, 76.5%	19.89 ± 1.42	Toronto Alexithymia Scale	Alexithymia and Social Media	TAS-20: 50.09 ± 8.22	As time spent on social media increases, alexithymia scores and difficulty in recognizing feelings also increase ($p < 0.05$).

Mohamed & Mostafa (2020), Egypt[9]	TC	320, 54.7%	NR	SAS, Hamilton Depression, Self-Esteem Inventory	Smartphone addiction, depression, and self-esteem	NR	Positive correlation with depression (p< 0.001) and negative correlation with self-esteem (p< 0.001)
Oh & Oh (2017), South Korea[49]	TC	329, 100%	NR	NISA Smartphone Addiction Proneness Scale; Rosenberg Self-Esteem Scale, IRI	Smartphone addiction, self-esteem, empathy, interpersonal relationships	NR	Negative correlations between the smartphone addiction and self-esteem (p <0.01) and showed significant correlations between the self-esteem and empathy (p <0.05).
Ozdil et al. (2022), Turkey[50]	TC	259, 80.7%	20.29 ± 1.60	SAS-SV, Numeric Rating Scale (NRS)	Smartphone addiction and musculoskeletal pain	SAS-SV: 25.71 ± 7.49	Association smartphone addiction with higher severity of headache, ear pain, shoulder pain and low back pain (p < 0.05)
Özer et al. (2023), Turkey[51]	TC	463, 74.7%	20.44 ± 1.46	IAS, CSS, DERS-16	Internet addiction, communication, emotional regulation	IAS: 31.38 ± 11.03; CSS: 91.71 ± 15.55; DERS-16: 44.09 ± 12.10	Internet addiction was negatively correlated with communication skills and positively with emotional regulation difficulties (p <0 .01).
Savci et al. (2021), Turkey[52]	TC	379, 76%	20.36 ± 1.17	SAS-SV, CLAS, CDMNS	Smartphone addiction and clinical decision-making	SAS-SV: 29.22 ± 9.89; CLAS: 49.60 ± 17.70; CDMNS: 139.20 ± 14.38	Smartphone addiction positively correlated with cyberloafing (r=0.42, p<0.01) and negatively correlated with clinical decision-making (r=-0.16, p<0.01).
Sok et al. (2019), South Korea[53]	TD	139, 84.2%	NR	Self-Control Scale, Daily Life Stress Scale, GICC	Self-control, stress, communication skills	NR	Nursing students in the smartphone addiction risk group had significantly lower self-control (p = 0.003) and higher daily life stress (p < 0.001) than the general group; no significant difference in communication skills
Sönmez et al. (2020), Turkey[54]	TC	682, 74.5%	20.76 ± 1.72	SAS-SV, UCLA Loneliness Scale	Smartphone addiction and loneliness	SAS-SV: 31.40 ± 10.17; UCLA: 40.31 ± 8.61	Positive correlation between smartphone addiction and loneliness (r = 0.169, p < 0.05)

Tárrega-Piquer et al. (2023), Spain[55]	TC	308, 88.6%	21.63 ± 5.24	NMP-Q, SAQ, APS-SF	Nomophobia, social anxiety, procrastination	–	Nomophobia affected 19.5%; NMP-Q was higher with more daily use and in-class checking (both p<0.001), inversely related to self-reported grades (p<0.001), not related to procrastination (p=0.114), and associated with social anxiety only bivariately (p<0.001; adjusted p=0.906).
Tastan et al. (2021), Turkey[20]	TC	333, 100%	20.24± 1.13	SAS-SV, IAS [Anxiousness]	Smartphone addiction and interaction anxiety	SAS-SV (stratified): 34.92 ± 10.38 (self-identified addicts) vs. 16.93 ± 6.69 (non-addicts).	Smartphone addiction correlated with higher social anxiety in interaction situations
Turan et al. (2020), Turkey[56]	TD	160, 93.1%	20.54 ± 1.90	IAS, UCLA, SWLS	Internet addiction, loneliness and life satisfaction	IAS: 33.76 ± 10.53; UCLA: 59.02 ± 8.21; SWLS: 22.72 ± 6.45	Internet addiction was at a moderate level; no significant correlation between internet addiction, loneliness, and life satisfaction (p > 0.05). A positive correlation was found between loneliness and life satisfaction (p < 0.05).
Turan et al. (2021), Turkey[57]	TD	518, 81%	20.72 ± 1.70	SMAS, CLS	Social media addiction and cyberloafing	SMAS: 71.30 ± 19.85; CLS: 87.71 ± 18.55	Moderate positive correlation between social media addiction and cyberloafing (p < 0.01)
Uzuncakmak et al. (2022), Turkey[58]	TC	771, 79.5%	20.89 ± 1.90	SAS-SV, PSQI, Epworth Sleepiness Scale	Smartphone addiction, sleep quality, daytime sleepiness	NR	High smartphone addiction related to poorer sleep quality and more daytime sleepiness (p <0.05)
Yaman Aktaş et al. (2022), Turkey[59]	TC	429, 76.2%	NR	DAS, Level 2-Sleep Disturbance	Digital addiction and sleep	DAS: 102.97 ± 24.30; Level2SD: 13.97 ± 3.92	Positive correlation between digital addiction and sleep disorders (r = 0.203, p < 0.01)
Yatmaz et al. (2022), Turkey[8]	TC	310, NR	NR	SAS, Life Goals Scale	Smartphone addiction and life goals	SAS: 100.23 ± 24.54	Significant relationship between mobile addiction and reduced life goal clarity

Zhao (2022), China[60]	TC	568, 87.7%	20.6 ± 1.33	FFMQ, LOT-R, Loneliness Scale, SDL Scale	Self-directed learning, mindfulness, optimism, loneliness	FFMQ: 117.65 ± 14.36; LOT-R: 15.96 ± 3.33; Loneliness: 40.61 ± 9.53; SDL: 139.08 ± 18.20	Mindfulness and optimism positively associated; loneliness negatively associated with SDL
Zhou et al. (2022), China[61]	TC	1445, NR	19.65 ± 1.35	SAS-SV, IPASN, ASES, ABS	Smartphone addiction, professional attitude, academic burnout	SAS-SV: 32.92 ± 8.05	Positive correlation between smartphone addiction and academic burnout ($p < 0.001$)

Note. Notes (instruments/acronyms): ABS = Academic Burnout Scale; ASES = Academic Self-Efficacy Scale; APS-SF = Academic Procrastination Scale–Short Form; AUDIT = Alcohol Use Disorders Identification Test; BPS = Bedtime Procrastination Scale; Care-Q = Caring Assessment Questionnaire; CDMNS = Clinical Decision-Making in Nursing Scale; CLAS = Cyberloafing Academic Scale; CLS = Cyberloafing Scale; CSS = Communication Skills Scale; CSAS = Communication Skills Assessment Scale; DAS = Digital Addiction Scale; DERS-16 = Difficulties in Emotion Regulation Scale (16 items); DGS = Daily Goals Scale; ESS = Epworth Sleepiness Scale; FFMQ = Five Facet Mindfulness Questionnaire; FoMO = Fear of Missing Out Scale; FPS = Future Perspective Scale; GICC = Global Interpersonal Communication Competence Scale; IAS = Internet Addiction Scale; IAS [Anxiousness] = Interaction Anxiousness Scale; ICCI = Interpersonal Communication Competence Inventory; ICQ / K-ICQ = Interpersonal Competence Questionnaire / Korean version; ImpSS = Impulsive Sensation Seeking Scale; IGDS9-SF = Internet Gaming Disorder Scale – Short Form; IPASN = Inventory of Professional Attitude for Student Nurses; IRI = Interpersonal Reactivity Index; ISI = Insomnia Severity Index; JSE = Jefferson Scale of Empathy; LOT-R = Life Orientation Test–Revised; MDMQ = Melbourne Decision Making Questionnaire; MMM = Media Multitasking Motivation; MPPUS / MPPUS-10 = Mobile Phone Problem Use Scale / 10-item short form; MSLQ = Motivated Strategies for Learning Questionnaire; MSPSS = Multidimensional Scale of Perceived Social Support; MTUAS = Media and Technology Usage and Attitudes Scale; NISA / SAP-NISA = Smartphone Addiction Proneness Scale (National Information Society Agency, Korea); NMP-Q = Nomophobia Questionnaire; NMQ = Nordic Musculoskeletal Questionnaire; NRS = Numeric Rating Scale (for pain); PB = Phubbing Behavior subscale (from the Phubbing Scale); PHQ-9 = Patient Health Questionnaire-9 (depression); PMPUS = Problematic Mobile Phone Use Scale; PRS = Peer Relations Scale; PSQI = Pittsburgh Sleep Quality Index; SAI / SPAI / SPAI-PV = Smartphone Addiction Inventory / Persian Version; SAQ / SAQ-A30 = Social Anxiety Questionnaire for Adults (30 items); SAS = Smartphone Addiction Scale (33 items); SAS-SV = Smartphone Addiction Scale–Short Version (10 items); SASp = Smartphone Addiction Scale–Proneness version; SDL Scale = Self-Directed Learning Scale; Self-Esteem Inventory / Rosenberg Self-Esteem = Rosenberg Self-Esteem Scale; SMAS / SMAS-AF = Social Media Addiction Scale / Adult Form; SQAPMPU = Short Questionnaire for Assessing Problematic Mobile Phone Use; SRF-S = Self-Regulatory Fatigue Scale–Short; SWLS = Satisfaction With Life Scale; TAS-20 = Toronto Alexithymia Scale–20; TEQ = Toronto Empathy Questionnaire; TIPI-C = Ten-Item Personality Inventory–Chinese

version; UCLA = UCLA Loneliness Scale; WANIS = WhatsApp Addiction and Negative Impact Scale (ad hoc, Gutiérrez-Puertas 2020); Level2SD = Level 2–Sleep Disturbance scale (DSM-5 Self-Rated Level 2 Cross-Cutting Symptom Measure).

TC = Transversal correlacional; TD = Transversal descriptivo; ED = Experimental design.*= Derived from reported data
