# Supplementary material for: Assessment Tools and Psychosocial Consequences of Smartphone Addiction in Nursing Students: A Systematic Review and Meta-Analysis
Source: Healthcare (Basel). 2025 Oct 20;13(20):2639. doi: 10.3390/healthcare13202639 (PMC12563757; doi:10.3390/healthcare13202639)
Supplement: Supplementary file 1 [file healthcare-13-02639-s001.zip › Supplementary Table S3.pdf]

Table S3 Critical appraisal of the included studies using the JBI Checklist for Analytical Cross-Sectional Studies.

Study	Q1. Inclusion criteria clearly defined	Q2. Subjects and setting described in detail	Q3. Exposure measured validly/reliably	Q4. Objective standard criteria used	Q5. Confounding factors identified	Q6. Strategies for confounding	Q7. Outcomes measured validly/reliably	Q8. Appropriate statistical analysis	Risk of Bias
Akturk & Budak (2019)[16]	Yes	Yes	Yes	Yes	Yes	No	Yes	Yes	Moderate
Alsayed et al. (2020)[3]	Yes	Yes	Yes	Yes	No	No	Yes	Yes	Moderate
Ayar & Günkan (2021)[17]	Yes	Yes	Yes	Yes	No	No	Yes	Yes	Moderate
Bajamal et al. (2023)[13]	Yes	Yes	Yes	Yes	Yes	Yes	Yes	Yes	Low
Barzegari et al. (2023)[23]	Yes	Yes	Yes	Yes	No	No	Yes	Yes	Moderate
Bayir & Topbas (2023)[22]	–	–	–	–	–	–	–	–	NA (Experimental)
Berdida & Grande (2023)[24]	Yes	Yes	Yes	Yes	No	No	Yes	Yes	Moderate
Bilgic et al. (2023)[10]	Yes	Yes	Yes	Yes	No	No	Yes	Yes	Moderate
Catiker et al. (2021)[25]	Yes	Yes	Yes	Yes	Unclear	No	Yes	Yes	Moderate

Study	Q1. Inclusion criteria clearly defined	Q2. Subjects and setting described in detail	Q3. Exposure measured validly/reliably	Q4. Objective standard criteria used	Q5. Confounding factors identified	Q6. Strategies for confounding	Q7. Outcomes measured validly/reliably	Q8. Appropriate statistical analysis	Risk of Bias
Çelik Ince (2021)[26]	Yes	Yes	Yes	Yes	No	No	Yes	Yes	Moderate
Celikkalp et al. (2020)[27]	Yes	Yes	Yes	Yes	No	No	Yes	Yes	Moderate
Cerit et al. (2018)[28]	Yes	Yes	Yes	Yes	Unclear	No	Yes	Yes	Moderate
Chen et al. (2022)[29]	Yes	Yes	Yes	Yes	Yes	Yes	Yes	Yes	Low
Cho & Lee (2016)[2]	Yes	Yes	Yes	Yes	No	No	Yes	Yes	Moderate
Çobanoğlu et al. (2021)[15]	Yes	Yes	Yes	Yes	Yes	No	Yes	Yes	Moderate
Dayapoglu et al (2016)[30]	Yes	Yes	Yes	Yes	No	No	Yes	Yes	Moderate
Demiralp et al. (2021)[12]	No	Yes	Yes	Yes	No	No	Yes	Yes	Moderate
El-Ashry et al. (2024)[31]	Yes	Yes	Yes	Yes	Yes	No	Yes	Yes	Moderate
Eskin Bakaksiz et al.	Yes	Yes	Yes	Yes	No	No	Yes	Yes	Moderate

Study	Q1. Inclusion criteria clearly defined	Q2. Subjects and setting described in detail	Q3. Exposure measured validly/reliably	Q4. Objective standard criteria used	Q5. Confounding factors identified	Q6. Strategies for confounding	Q7. Outcomes measured validly/reliably	Q8. Appropriate statistical analysis	Risk of Bias
(2022)[32]									
Ghosh et al. (2021)[7]	Yes	Yes	Yes	Yes	No	No	Yes	Yes	Moderate
Gutiérrez-Puertas et al. (2020)[33]	Yes	Yes	Yes	Yes	No	No	Yes	Yes	Moderate
Gutiérrez-Puertas et al. (2019)[34]	Yes	Yes	Yes	Yes	No	No	Yes	Yes	Moderate
Han et al. (2022)[35]	Yes	Yes	Yes	Yes	Yes	No	Yes	Yes	Moderate
Ilter & Ovayolu (2022)[36]	Yes	Yes	Yes	Yes	Yes	No	Yes	Yes	Moderate
Jose et al. (2024)[37]	Yes	Yes	Yes	Yes	No	No	Yes	Yes	Moderate
Kalal et al. (2023)[1]	Yes	Yes	Yes	Yes	No	No	Yes	Yes	Moderate
Kargin et al. (2020)[38]	Yes	Yes	Yes	Yes	Yes	No	Yes	Yes	Moderate
Khatgaonkar et al. (2020)[39]	Yes	Yes	No	No	No	No	No	No	High

Study	Q1. Inclusion criteria clearly defined	Q2. Subjects and setting described in detail	Q3. Exposure measured validly/reliably	Q4. Objective standard criteria used	Q5. Confounding factors identified	Q6. Strategies for confounding	Q7. Outcomes measured validly/reliably	Q8. Appropriate statistical analysis	Risk of Bias
Lee et al. (2018)[40]	Yes	Yes	Yes	Yes	Yes	No	Yes	Yes	Moderate
Lee et al. (2022)[41]	Yes	Yes	Yes	Yes	No	No	Yes	Yes	Moderate
Lobo et al. (2022)[42]	Yes	Yes	Yes	Yes	Yes	No	Yes	Yes	Moderate
Machado et al. (2023)[43]	Yes	Yes	Yes	Yes	No	No	Yes	Yes	Moderate
Mancheri et al. (2023)[44]	Yes	Yes	Yes	Yes	Yes	No	Yes	Yes	Moderate
Marletta et al. (2021)[45]	Yes	Yes	Yes	Yes	No	No	Yes	Yes	Moderate
Márquez-Hernández et al. (2020)[46]	Yes	Yes	Yes	Yes	Yes	No	Yes	Yes	Moderate
Mersal et al. (2024)[47]	Yes	Yes	Yes	Yes	Yes	Yes	Yes	Yes	Low
Mersin et al. (2020)[48]	Yes	Yes	Yes	Yes	No	No	Yes	Yes	Moderate
Mohamed &	Yes	Yes	Yes	Yes	No	No	Yes	Yes	Moderate

Study	Q1. Inclusion criteria clearly defined	Q2. Subjects and setting described in detail	Q3. Exposure measured validly/reliably	Q4. Objective standard criteria used	Q5. Confounding factors identified	Q6. Strategies for confounding	Q7. Outcomes measured validly/reliably	Q8. Appropriate statistical analysis	Risk of Bias
Mostafa (2020)[9]									
Oh & Oh (2017)[49]	Yes	Yes	Unclear	Unclear	Yes	Yes	Unclear	Yes	High
Ozdil et al. (2022)[50]	Yes	Yes	Yes	Yes	No	No	Yes	Yes	Moderate
Özer et al. (2023)[51]	Yes	Yes	Yes	Yes	Yes	No	Yes	Yes	Moderate
Savci et al. (2021)[52]	Yes	Yes	Yes	Yes	Yes	Yes	Yes	Yes	Low
Sok et al. (2019)[53]	Yes	Yes	Yes	Yes	Yes	No	Yes	Yes	Moderate
Sönmez et al. (2020)[54]	Yes	Yes	Yes	Yes	No	No	Yes	Yes	Moderate
Tárrega-Piquer et al. (2023)[55]	Yes	Yes	Yes	Yes	Yes	No	Yes	Yes	Moderate
Tastan et al. (2021)[20]	Yes	Yes	Yes	Yes	No	No	Yes	Yes	Moderate
Turan et al. (2020)[56]	Yes	Yes	Yes	Yes	No	No	Yes	Yes	Moderate

Study	Q1. Inclusion criteria clearly defined	Q2. Subjects and setting described in detail	Q3. Exposure measured validly/reliably	Q4. Objective standard criteria used	Q5. Confounding factors identified	Q6. Strategies for confounding	Q7. Outcomes measured validly/reliably	Q8. Appropriate statistical analysis	Risk of Bias
Turan et al. (2021)[57]	Yes	Yes	Yes	Yes	No	No	Yes	Yes	Moderate
Uzuncakmak et al. (2022)[58]	Yes	Yes	Yes	Yes	Yes	Yes	Yes	Yes	Low
Yaman Aktas at al. (2022)[59]	Yes	Yes	Yes	Yes	No	No	Yes	Yes	Moderate
Yatmaz et al. (2022)[8]	Yes	Yes	Yes	Yes	Yes	No	Yes	Yes	Moderate
Zhao (2022)[60]	Yes	Yes	Yes	Yes	Yes	Yes	Yes	Yes	Low
Zhou et al. (2022)[61]	Yes	Yes	Yes	Yes	Yes	Yes	Yes	Yes	Low

Note: "Yes" indicates the criterion was met; "No" indicates it was not met; "Unclear" means information was insufficient. JBI = Joanna Briggs Institute.
